# Dietary Iron Supplementation Alters Hepatic Inflammation in a Rat Model of Nonalcoholic Steatohepatitis

**DOI:** 10.3390/nu10020175

**Published:** 2018-02-04

**Authors:** Machi Atarashi, Takeshi Izawa, Rena Miyagi, Shoko Ohji, Ai Hashimoto, Mitsuru Kuwamura, Jyoji Yamate

**Affiliations:** Laboratory of Veterinary Pathology, Osaka Prefecture University, 1-58 Rinku Orai Kita, Osaka 598-8531, Japan; blue.ocean-orca-@live.jp (M.A.); rena.miyagi@gmail.com (R.M.); swc01002@edu.osakafu-u.ac.jp (S.O.); gure72@gmail.com (A.H.); kuwamura@vet.osakafu-u.ac.jp (M.K.); yamate@vet.osakafu-u.ac.jp (J.Y.)

**Keywords:** dietary iron supplementation, macrophage, nonalcoholic steatohepatitis (NASH)

## Abstract

Nonalcoholic fatty liver disease (NAFLD) is now the most common liver disease in the world. NAFLD can progress to nonalcoholic steatohepatitis (NASH), cirrhosis and eventually hepatocellular carcinoma. Acquired hepatic iron overload is seen in a number of patients with NAFLD; however, its significance in the pathology of NAFLD is still debated. Here, we investigated the role of dietary iron supplementation in experimental steatohepatitis in rats. Rats were fed a control, high-fat (HF), high-fat high-iron (HFHI) and high-iron (HI) diet for 30 weeks. Blood biochemical, histopathological and gut microbiota analyses were performed. Rats in HF and HFHI groups showed an ALT-dominant elevation of serum transaminases, hepatic steatosis, hepatic inflammation, and upregulation of proinflammatory cytokines. The number of large inflammatory foci, corresponding to lobular inflammation in NASH patients, was significantly higher in HFHI than in HF group; within the lesion, macrophages with intense iron staining were observed. Hepatic expression of TNFα was higher in HFHI than that in HF group. There was no significant change in hepatic oxidative stress, gut microbiota or serum endotoxin levels between HF and HFHI groups. These results suggested that dietary iron supplementation enhances experimental steatohepatitis induced by long-term high-fat diet feeding in rats. Iron-laden macrophages can play an important role in the enhancement of hepatic inflammation.

## 1. Introduction

Chronic liver disease (CLD) is an intractable disease that can progress to cirrhosis and eventually hepatocellular carcinoma (HCC). HCC is the third leading cause of cancer death in the world [1]. The major causes of CLD are viral hepatitis (hepatitis C and B), alcoholic liver disease and nonalcoholic fatty liver disease (NAFLD); the prevalence of NAFLD has been rapidly increasing, and NAFLD has become the most common cause of CLD [2]. NAFLD progresses to HCC very slowly, which causes delayed detection of its advance [3]. 

Iron overload is a condition of excess body iron, which leads to iron accumulation in organs such as the liver, heart, pancreas and endocrine glands, resulting in dysfunctions of these vital organs [4]. In healthy individuals, hepatocytes have a large capacity for iron storage [5]. Hepatocytes also produce hepcidin, a central regulator of iron homeostasis. It negatively regulates iron efflux by internalization and subsequent degradation of ferroportin 1, the iron exporter located on the surface of duodenal enterocytes, macrophages and hepatocytes. However, when persistent liver injury occurs, hepcidin production is decreased, thereby leading to secondary iron overload [6,7]. Increased hepatic iron accumulation can contribute to additional liver injury via production of reactive oxygen species (ROS) [8]. Therefore, iron overload occurring in CLD is considered as an important risk factor for the progression of the disease [6,9]. 

Hepatic iron overload occurs in a variety of CLD. It is found in more than one third of patients with chronic hepatitis (types C and B) and NAFLD [8,10,11]. Additionally, primary iron overload occurs in patients with hereditary hemochromatosis, a genetic syndrome caused by mutations in any gene of the hepcidin-ferroportin axis [4]. Hereditary hemochromatosis is characterized by excessive iron accumulation in the liver, leading to direct liver injury and slow progression of cirrhosis and hepatocellular carcinoma. Several studies have suggested that heterozygosity for *HFE* gene mutation, the most common mutation type of hereditary hemochromatosis, can exacerbate chronic liver diseases, presumably via increased iron accumulation [12,13,14]; however, the role of *HFE* mutation in the pathology of NAFLD and chronic hepatitis C has not been confirmed [15,16,17,18]. 

Recent studies have reported that iron accumulation in hepatic reticuloendothelial system (RES) cells is associated with increased apoptosis and nonalcoholic steatohepatitis (NASH) pathology in NAFLD [8,19]. In contrast, other studies have suggested that iron burden does not contribute significantly to hepatic fibrosis in the majority of NAFLD patients [20,21]. Besides, the impact of iron depletion treatment by phlebotomy is still controversial in NAFLD [22,23]. Therefore, the pathophysiological role of iron overload in NASH remains to be elucidated.

In this study, we investigated the pathological role of dietary iron supplementation using a rat long-term model of nonalcoholic steatohepatitis. 

## 2. Materials and Methods 

### 2.1. Animals

Six-week-old male F344/DuCrlCrlj rats (Charles River Laboratories Japan, Yokohama, Japan) were divided into control (cont), high-fat (HF), high-fat high-iron (HFHI) and high-iron (HI) groups. Rats of each group were fed a diet listed in Table 1 for 30 weeks. The HF diet was selected from a recent study of murine NASH model [24], with a minor modification. Control rats were fed a standard diet (CE-2; CLEA Japan, Tokyo, Japan). All diets were purchased from CLEA Japan. Food and water were provided *ad libitum*. Rats were deeply anesthetized by isoflurane, and blood and liver were collected at weeks 5, 10, 20 and 30. Control rats were sampled at weeks 5 and 30. Four rats were analyzed from each group at each time point, except HFHI group at week 30 (3 rats). Fasting was performed overnight before each sampling. All animal experiments and were approved by the Animal Care and Use Committee of Osaka Prefecture University (code No. 25-1 and 27-19) were performed according to the institutional guidelines for animal experimentation.

### 2.2. Biochemical Analyses

Blood from the abdominal aorta was separated by centrifugation (3000 rpm, 4 °C, 5 min) and serum was collected. Blood biochemical and liver iron analyses were performed in SRL Inc. (Tokyo, Japan) as previously described [25]. Briefly, liver tissues were homogenized in a mixed solution of nitric acid and hydrogen peroxide and lysed by microwave irradiation. The solution was used for determining hepatic iron content by atomic absorption spectrometry. Hepatic malondialdehyde content was measured by thiobarbituric acid reacting substances (TBARS) method (Nikken SEIL Co., Ltd., Shizuoka, Japan). Serum endotoxin (lipopolysaccharide; LPS) was measured by limulus amebocyte lysate method (Seikagaku Corp., Tokyo, Japan).

### 2.3. Histopathology

The left lateral lobe of the liver was fixed in 10% neutral-buffered formalin, routinely processed, embedded in paraffin, cut at 5 µm and stained with hematoxylin and eosin (HE) for histopathological examination and with 3,3′-diaminobenzidine (DAB)-enhanced Perls stain for detecting iron deposition as previously described [25].

### 2.4. Cell Counts

The number of microgranulomas and large inflammatory foci were counted in the liver parenchyma. Five different fields from three animals were evaluated.

### 2.5. Real-Time Reverse Transcriptase Polymerase Chain Reaction (RT-PCR)

Liver samples were immersed in RNAlater regent (Qiagen, Hilden, Germany) and stored at −80 °C before use. Samples at weeks 20 and 30, the period during which marked steatohepatitis occurs in HF and HFHI groups, were used for cytokine expression analysis. For controls, samples at weeks 5 and 30 were used. Total RNA was extracted using SV Total RNA Isolation System (Promega, WI, USA). Real-time RT-PCR was performed with Thunderbird Probe qPCR Mix (Toyobo, Osaka, Japan) and TaqMan Gene Expression Assays (Life Technologies, CA, USA) in PicoReal 96 Real-time PCR system (Thermo Scientific, Waltham, MA, USA) as previously described [25,26,27]. The probe and primer sets are listed in Table 2. The data were analyzed using the comparative Ct method. 18S rRNA was used as an internal control.

### 2.6. Gut Microbiota Analysis

Bacterial DNA from cecal content was extracted by Mora Extract kit (Kyokuto Pharmaceutical Industrial Co., Ltd., Tokyo, Japan) according to the manufacturer’s instruction. The V3-V4 region of bacterial 16S rRNA gene was amplified by conventional PCR with KAPA HiFi HotStart ReadyMix (KAPA Biosystems, MA, USA) and a specific primer set (forward; 5′-TCGTCGGCAGCGTCAGATGTGTATAAGAGACAGCCTACGGGNGGCWGCAG-3′, reverse; 5′-GTCTCGTGGGCTCGGAGATGTGTATAAGAGACAGGACTACHVGGGTATCTAATCC-3′). The amplicons were then purified by AMPure XP (Beckman Coulter, CA, USA). Sequencing was performed on a MiSeq instrument (Illumina, CA, USA). Paired-end reads were merged and processed using MacQIIME software v1.9.1 (http://www.wernerlab.org/software/macqiime). The data processing included read error correction, chimera filtering, de novo picking of operational taxonomic units at a 97% identity cutoff, and assignment of taxonomic information.

### 2.7. Statistical Analysis

Data are presented as mean ± SD. Statistical analyses were performed using Prism software v5.0d (Graphpad, CA, USA) with Tukey–Kramer’s or Bonferroni’s multiple comparison. A value of *p* < 0.05 was considered statistically significant. Correlation analysis was performed by Pearson’s correlation coefficients.

## 3. Results

### 3.1. Dietary Iron Burden Enhances High-Fat Diet-Induced Hepatic Inflammation

#### 3.1.1. Clinical and Gross Findings

**Figure 1:**
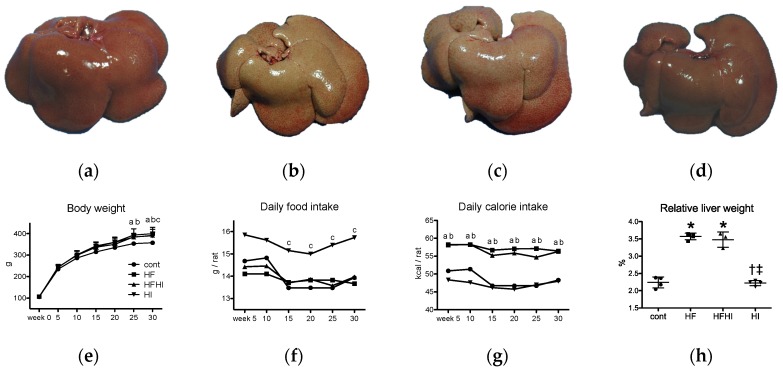
Gross images of the liver of (**a**) cont; (**b**) high-fat (HF); (**c**) high-fat and high-iron (HFHI) and (**d**) high-iron (HI) group at week 30. Temporal changes of (**e**) body weight; (**f**) daily food intake and (**g**) daily calorie intake (*n* = 4 or 3 in each group). ^a^ *p* < 0.05, HF vs. cont, ^b^ HFHI vs. cont, ^c^ HI vs. cont, by Bonferroni’s multiple comparison. (**h**) Liver relative weight data at week 30. * *p* < 0.05 vs. control, ^†^ vs. HF, ^‡^ vs. HFHI, by Tukey-Kramer’s multiple comparison.

Grossly, the livers of HF and HFHI groups were pale and remarkably enlarged compared with control livers (Figure 1a–c); both relative and absolute liver weights increased significantly in HF and HFHI groups (Figure 1h). The livers of the HI group were brownish in color and similar in size and weight compared with control livers (Figure 1a,d,h). Body weight of the HF and HFHI groups increased significantly at weeks 20 and 30, while that in the HI group increased significantly at week 30 (Figure 1e). Daily food intake was similar between cont, HF and HFHI groups, and higher in HI group (Figure 1f). Daily calorie intake, calculated by multiplication of daily food intake (Figure 1f) and caloric value of each diet (Table 1), was higher in HF and HFHI groups (Figure 1g).

#### 3.1.2. Blood Biochemical Findings

**Figure 2:**
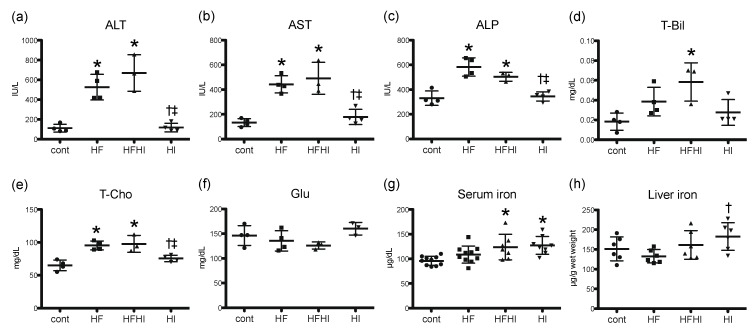
Blood biochemical data of (**a**) alanine aminotransferase (ALT); (**b**) aspartate aminotransferase (AST); (**c**) alkaline phosphatase (ALP); (**d**) total bilirubin; (**e**) total cholesterol; (**f**) glucose; (**g**) serum iron and (**h**) liver iron (**a**–**f**: *n* = 4 or 3, **g**: *n* = 7 to 10, **h**: *n* = 6 in each group). Data are expressed as mean ± standard deviation (SD). * *p* < 0.05 vs. control, ^†^ vs. HF, ^‡^ vs. HFHI, by Tukey-Kramer’s multiple comparison.

Blood biochemical analyses showed a significant increase in alanine aminotransferase (ALT), aspartate aminotransferase (AST), alkaline phosphatase (ALP) and total cholesterol (T-cho) in HF and HFHI groups at week 30 (Figure 2a–c,e). In HFHI group, total bilirubin (T-Bil) also increased significantly (Figure 2d). These parameters did not change in HI group (Figure 2a–e). Blood glucose did not differ between all groups (Figure 2f). Serum iron increased significantly in HFHI and HI groups (Figure 2g), and liver iron increased significantly in HI group (Figure 2h). Details of blood biochemical data, including data at all time points, are listed in Table S1. ALT-dominant elevation of serum transaminases with marked hepatomegaly was seen from week 20 (Figure S1).

#### 3.1.3. Histopathological Findings

**Figure 3:**
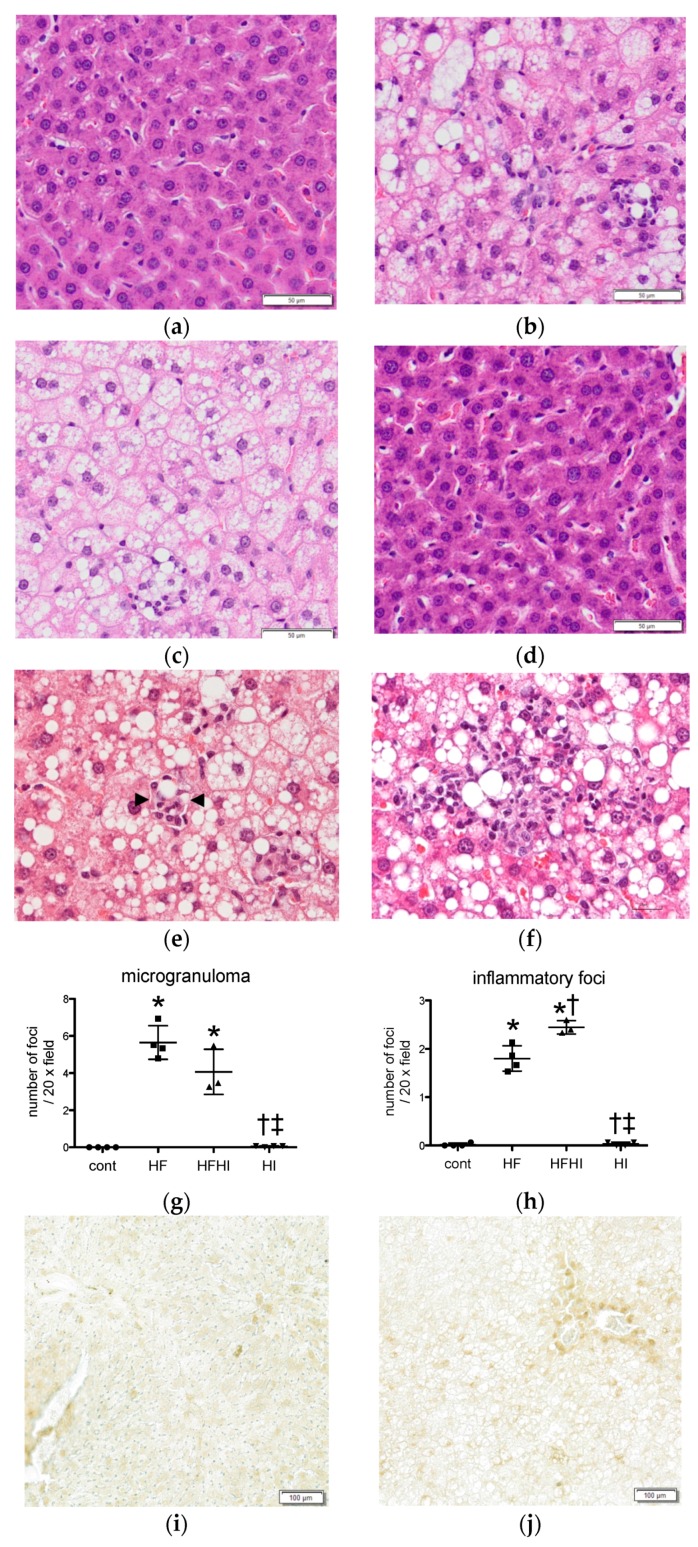

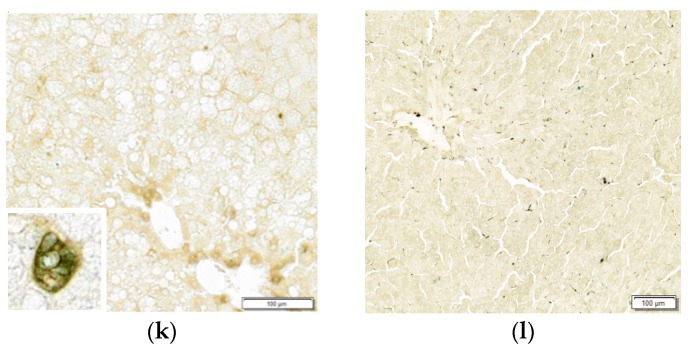
Histopathology of the liver in (**a**) cont; (**b**) high-fat (HF); (**c**) high-fat and high iron (HFHI) and (**d**) high-iron (HI) groups at week 30. Hematoxylin and eosin (HE) stain, bar: 50 µm. In HF and HFHI groups, (**e**) microgranuloma (surrounded by arrowheads) and (**f**) large inflammatory foci are seen in the hepatic parenchyma. HE stain, bar: 20 µm. The number of (**g**) microgranulomas and (**h**) inflammatory foci per 20× field were counted (*n* = 4 or 3 in each group). * *p* < 0.05 vs. control, ^†^ vs. HF, ^‡^ vs. HFHI, by Tukey–Kramer’s multiple comparison. Iron staining of the liver in (**i**) cont; (**j**) HF; (**k**) HFHI and (**l**) HI groups. DAB-enhanced Perls stain, bar: 100 µm. Inset in (**k**) indicates a microgranuloma with intense iron staining.

Histopathologically, lipid accumulation and swelling of hepatocytes (hepatic steatosis) were observed in HF and HFHI groups compared with control liver (Figure 3a–c). The livers of HI group were almost normal, except for the presence of eosinophilic cytoplasmic fine granules in hepatocytes, suggestive of cytoplasmic iron stores (Figure 3d). Two different types of inflammatory lesions were seen in HF and HFHI groups; (1) small aggregates of macrophages phagocytizing degenerative or necrotic hepatocytes (microgranuloma; Figure 3e, surrounded by arrowheads) and (2) large foci consisting of macrophages, lymphocytes and neutrophils (inflammatory foci; Figure 3f), corresponding to lobular inflammation seen in NAFLD patients. The number of microgranulomas and large inflammatory foci increased significantly in HF and HFHI groups (Figure 3g,h). In HFHI group, the number of inflammatory foci was higher than that in HF group at week 30 (Figure 3h). Iron histochemistry revealed an iron accumulation in hepatocytes and macrophages in HI group (Figure 3i–l). In HFHI group, intense iron staining was seen in macrophages, especially macrophages constituting microgranulomas (Figure 3k, inset).

#### 3.1.4. Expression Patterns of Inflammatory Cytokines

**Figure 4:**
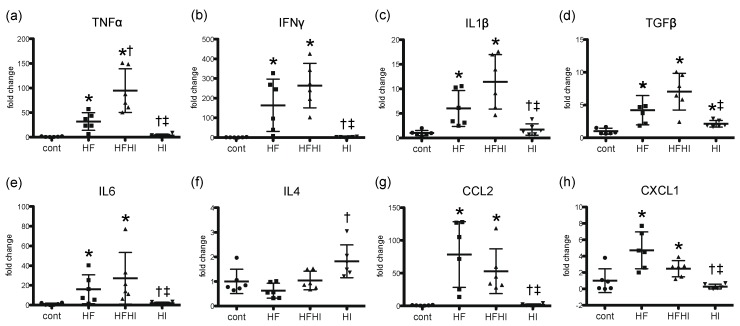
Hepatic expression of inflammatory cytokine and chemokine genes at weeks 20 and 30 (*n* = 6 in each group). (**a**) *TNFα*; (**b**) *IFNγ*; (**c**) *IL1β*; (**d**) *TGFβ*; (**e**) *IL6*; (**f**) *IL4*; (**g**) *CCL2* and (**h**) *CXCL1*. Data were normalized to *18s rRNA* and are expressed as fold change from control (mean ± standard deviation). * *p* < 0.05 vs. control, ^†^ vs. HF, ^‡^ vs. HFHI, by Tukey-Kramer’s multiple comparison.

To investigate molecular changes in hepatic inflammation, hepatic expression of inflammatory cytokines was analyzed by real-time PCR. Expression of *TNFα*, *IFNγ*, *IL1β*, *TGFβ*, *IL6*, *CCL2* and *CXCL1* mRNA significantly increased in HF and HFHI groups compared with control and HI groups at weeks 20 and 30 (Figure 4a–e,g,h); expression levels of *TNFα* mRNA were significantly higher in HFHI than that in HF group (Figure 4a). Expression of *IL4* mRNA did not change significantly in HF and HFHI groups (Figure 4f).

### 3.2. Changes in Oxidative Stress-Related Molecules 

**Figure 5:**
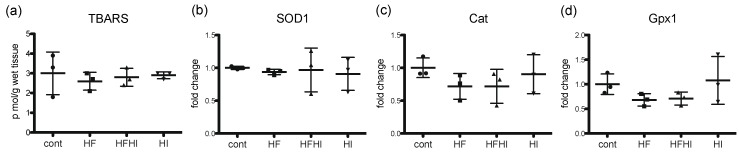
(**a**) Hepatic TBARS levels at week 30. Hepatic expression of antioxidant enzyme genes; (**b**) superoxide dismutase 1 (*SOD1*); (**c**) catalase (*Cat*) and (**d**) glutathione peroxidase 1 (*Gpx1*) (*n* = 3 in each group). Data were normalized to 18S rRNA and are expressed as fold change from control (mean ± SD). Data were statistically analyzed by Tukey–Kramer’s multiple comparison. HF, high-fat; HFHI, high-fat and high-iron; HI, high-iron.

To investigate the oxidative stress condition in the liver, hepatic TBARS levels, an end product of lipid peroxidation, and expression of antioxidant enzyme genes were analyzed. There were no significant changes in hepatic TBARS levels and expression of *SOD1*, *Cat* and *Gpx1* mRNA between all the groups at week 30 (Figure 5).

### 3.3. Changes in Gut Microbiota

**Figure 6:**
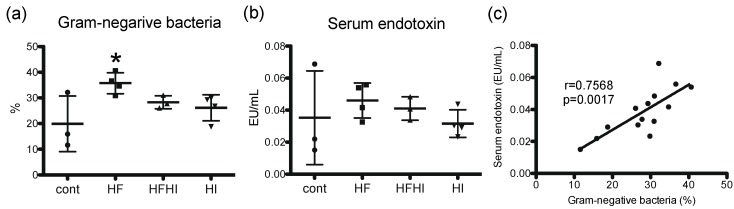
(**a**) Abundance of Gram-negative bacteria in the gut and (**b**) serum endotoxin level at week 30 (*n* = 4 or 3 in each group). * *p* < 0.05 vs. control, by Tukey–Kramer’s multiple comparison. (**c**) Correlation analysis between gut Gram-negative bacteria and serum endotoxin. Data were analyzed by Pearson’s correlation. HF, high-fat; HFHI, high-fat and high-iron; HI, high-iron.

To investigate whether gut dysbiosis is involved in NASH pathology of this model, gut microbiota was analyzed by sequencing bacterial *16S rRNA* gene using MiSeq. Compared with the control group, abundance of Gram-negative bacteria increased significantly in the HF group, and did not change in HFHI and HI groups at week 30 (Figure 6a). Serum endotoxin level did not differ significantly between all groups at week 30 (Figure 6b). Abundance of Gram-negative bacteria was positively correlated with serum endotoxin level (Figure 6c).

**Figure 7:**
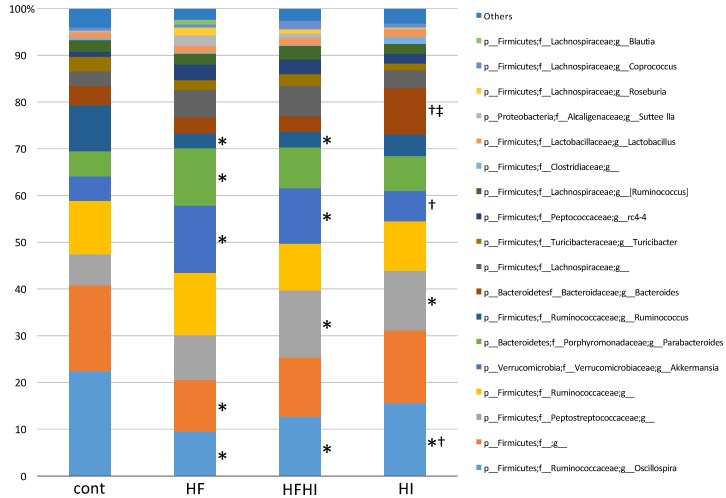
Taxonomic composition of gut microbiota at week 30. * *p* < 0.05 vs. cont, ^†^ vs. HF, ^‡^ vs. HFHI by Bonferroni’s multiple comparison. HF, high-fat; HFHI, high-fat and high-iron; HI, high-iron.

Taxonomic composition and abundance of the gut microbiota are shown in Figure 7 and Table 3, respectively. Abundance of genera *Oscillospira* and *Ruminococcus* decreased significantly and abundance of genus *Akkermansia* increased significantly in HF and HFHI groups, compared with control group at week 30. In addition, abundance of family Peptostreptococcaceae increased significantly in HFHI and HI groups.

## 4. Discussion

Our results showed that the large inflammatory foci, corresponding to lobular inflammation in NASH patients, increased in HFHI group compared to HF group, suggesting an enhancement of hepatic inflammation by dietary iron supplementation. The role of iron accumulation in CLD has been studied in human NASH cases [28]. Increased stainable hepatic iron or biochemical hepatic iron content is associated with more advanced fibrosis in NASH patients [29]. Expression of heme oxygenase-1, a gene induced during oxidative stress, is positively correlated with hepatic TBARS levels, a marker for lipid peroxidation, in NAFLD patients [30]. Oxidative stress is the well-known mechanism for iron-induced hepatotoxicity, although the hepatic TBARS levels did not change in our model. During hepatic iron overload, Fe^2+^, a labile form of iron, increases in the blood as non-transferrin-bound iron (NTBI) [31]. Then, the labile plasma iron is taken up by hepatocytes and enters the cytosolic iron pool, stimulating production of ROS such as hydroxyl radicals by Fenton reaction. Generation of such highly-reactive ROS can damage cellular macromolecules, leading to lipid peroxidation. Lipid peroxidation by-products can further induce damage to mitochondria, plasma membrane and DNA, resulting in cell injury or death. In NASH patients with iron overload, the modified guanosine base 8-hydroxydeoxyguanosine levels, a marker for oxidatively generated DNA damage, are positively correlated to histologic total iron score [32]. These data suggest that iron-induced oxidative stress can be involved in the progression of NASH pathology. However, in our model, the hepatic oxidative stress did not change at week 30, despite the evidence of moderate to advanced steatohepatitis. One possibility to explain this discrepancy is that oxidative stress might increase at the earlier time point, involved in the early disease onset. Another possibility is that iron accumulation can enhance hepatic inflammation by the mechanism other than oxidative stress.

We showed an intense iron deposition in the sinusoidal macrophages consisting of microgranuloma, suggesting an important role of macrophages/Kupffer cells in the iron-induced enhancement of hepatic inflammation. Interestingly, the expression levels of *TNFα* were significantly higher in HFHI than those in HF group, suggesting an important role of TNFα in the extension of hepatic inflammation in the NASH model. In NAFLD patients, iron deposition of RES cells is associated with NASH, increased apoptosis, and increased oxidative stress [8,19]. Macrophages/Kupffer cells, the main RES constituent, are the key effector cells in hepatic inflammation, and a major source of TNFα. TNFα promotes recruitment of neutrophils and other inflammatory cells, and induces cytotoxicity such as apoptosis [33]. In vitro studies have shown that iron treatment on cultured hepatic macrophages increases TNFα expression in a nuclear factor-kappa B-dependent manner, which is abrogated by iron chelate treatment [34,35,36]. The expression levels of *IFNγ*, *IL1β* and *TGFβ* did not change significantly, but tended to be higher (approximately 1.5- to 2-fold) in HFHI than in HF group. Since the sample size was relatively small in this study, the possibility of a type II error cannot be ruled out. Further study with a larger sample size would detect a statistically significant increase of such cytokines. Taken together, the sinusoidal macrophages with iron accumulation in our NASH model may be capable of augmenting inflammation, presumably by overproducing proinflammatory cytokines.

LPS is a well-known activator of Kupffer cells, which is recognized by Toll-like receptor 4, leading to nuclear factor κB-induced proinflammatory cytokine production. In NASH patients, serum LPS levels are elevated and are associated with gut dysbiosis [37]. However, in this study, there is no significant change in total abundance of Gram-negative bacteria in the gut or serum LPS levels between HF and HFHI groups, suggesting that changes in gut microbiota are not involved in the enhancement of hepatic inflammation by dietary iron supplementation.

*Oscillospira* bacteria are considered to have an important role in lipid homeostasis, as its abundance is positively associated with hepatic cholesterol and phospholipid in mice [38]. Decreased abundance of *Oscillospira* in the gut has been reported in NASH patients and NAFLD model mice [39,40]. Therefore, the decreased abundance of genus *Oscillospira* may indicate a disruption of lipid homeostasis. *Ruminococcus* abundance was shown to be positively associated with hepatic fibrosis in NASH patients [41]. In this study, the *Ruminococcus* abundance was significantly reduced by high-fat diet feeding; it may be unrelated to fibrosis as the fibrosis was mild at week 30. Abundance of *Akkermansia*, particularly *Akkermansia muciniphila*, has been recently reported to increase in type 2 diabetes patients and NAFLD model rats [39,42]. Other studies have suggested that *Akkermansia* has a role in reducing plasma LPS level and body fat accumulation [43,44]. Therefore, the increased abundance of *Akkermansia* in our model might be a protective reaction to NAFLD condition. The Peptostreptococcaceae family, such as *Peptostreptococcus asaccharolyticus*, are bacteria that can synthesize iron-sulfur proteins [45]. The increased abundance of Peptostreptococcaceae by high-iron diet feeding in this study may be compensatory response to iron overload in the gut.

It is well known that the high-fat diet feeding in rodents can induce liver disease similar to human NAFLD/NASH; however, excessive intake of carbohydrates and diabetes are a more important basis of NAFLD development in humans. A recent study demonstrated that long-term feeding of a low-fat/high-carbohydrate diet in mice induces hepatic cancer based on the NAFLD/NASH-like liver disease [46]. Besides, the Western diet model (high-fat/high-carbohydrate diet with sugar water drinking) has recently been used frequently for NAFLD/NASH study, as it can mimic the physiological, metabolic, histological, transcriptomic features and clinical endpoints of human NASH [47]. Therefore, addition of high carbohydrates to our model would be a better choice to produce a more relevant NASH model.

In this study, the supplementation of the diet with 0.5% iron had only a modest effect (1.2-fold increase) on liver iron content, which was an unexpected finding. Approximately 75 mg per day of iron was ingested from the diet in rats with HFHI and HI groups, which is more than the upper limit (45 mg) of iron intake in adult humans [48]. Thus, the iron supplementation in this study was not exaggerated, but rather need to be increased to induce hepatic iron overload. The dietary iron content in the present study was determined by our preliminary data of four-week feeding study of a 0.5% iron diet, showing a two-fold increase in serum and liver iron content in the same rat strain (unpublished data). The cause of this dissociation has not been specified; however, background diet (i.e., the source of carbohydrate and fibers) could be an important factor, as it is the only difference of the experimental protocol between the two studies. The study of feeding the same high-fat diet containing 1% iron on rats is now in progress.

## 5. Conclusions 

This study suggested that dietary iron supplementation enhances experimental steatohepatitis induced by long-term high-fat diet feeding in rats. Iron-laden macrophages, likely in the active inflammatory state, can be responsible for the enhancement of hepatic inflammation.

## Figures and Tables

**Table 1:** Diet composition.

Diet	Cont	HF	HFHI	HI
CE-2 * (g %)	100	88	85	97
cocoa butter (g %)	0	11	11	0
cholesterol (g %)	0	1	1	0
iron (III) citrate (g %)	0	0	2.9	2.9
iron content (g %)	0.03	0.03	0.50	0.51
protein (g %)	24.7	21.7	21.0	21.7
fat (g %)	4.8	16.2	16.0	4.2
carbohydrate (g %)	51.3	45.1	43.6	45.1
protein (kcal %)	28.5	21.0	20.9	28.5
fat (kcal %)	12.3	35.3	35.8	12.3
carbohydrate (kcal %)	59.2	43.7	43.3	59.2
total calories (kcal/100 g diet)	346.5	412.9	402.9	309.4

HF: high fat, HFHI: high fat and high iron, HI: high iron. * CE-2: standard rodent diet, containing wheat, corn, milo as the source of carbohydrates.

**Table 2:** Polymerase Chain Reaction (PCR) probes used in this study.

Gene Name	Abbreviation	Probe Assay ID
Tumor necrosis factor-α	TNFα	Rn01525859_g1
Interferon-γ	IFNγ	Rn00594078_m1
Interleukin-1β	IL1β	Rn00580432_m1
Transforming growth factor-β	TGFβ	Rn00572010_m1
Interleukin-6	IL6	Rn01410330_m1
Interleukin-4	IL4	Rn01456866_m1
Chemokine (C-X-C motif) ligand 1	CXCL1	Rn00578225_m1
Chemokine (C-C motif) ligand 2	CCL2	Rn00580555_m1
Superoxide dismutase 1, soluble	SOD1	Rn00566938_m1
Catalase	Cat	Rn00560930_m1
Glutathione peroxidase 1	GPx1	Rn00577994_g1
18S ribosomal RNA	18S rRNA	Hs99999901_s1

**Table 3:** Abundance of gut microbiota at week 30.

Bacteria	Cont	HF	HFHI	HI
**Bacteroidetes**	**9.7**	**15.9**	**12.3**	**17.4**
Bacteroidaceae	4.1	3.5	3.4	9.9
*Bacteroides*	*4.1*	*3.5*	*3.4*	*9.9*^ †,‡^
Porphyromonadaceae	5.6	12.3	8.9	7.5
*Parabacteroides*	*5.6*	*12.3* *	*8.9*	*7.5*
**Firmicutes**	**84.3**	**67.3**	**74.5**	**75.5**
Clostridiaceae	0.9	0.6	0.5	1.6
Lachnospiraceae	6.9	12.0	12.4	7.3
*Blautia*	*0.0*	*1.0*	*0.0*	*0.0*
*Coprococcus*	*0.5*	*0.7*	*1.8*	*0.8*
*Roseburia*	*0.1*	*1.7*	*0.9*	*0.2*
Lactobacillaceae	1.2	1.4	1.6	1.7
*Lactobacillus*	*1.2*	*1.4*	*1.6*	*1.7*
Peptococcaceae	1.2	3.3	3.3	2.3
Peptostreptococcaceae	6.5	9.8	14.3 *	12.7 *
Ruminococcaceae	44.1	26.2	26.2	30.9
*Oscillospira*	*22.5*	*9.6* *	*12.7* *	*15.5* *^,†^
*Ruminococcus*	*9.8*	*3.2* *	*3.3* *	*4.6*
Turicibacteraceae	3.1	2.2	2.5	1.6
*Turicibacter*	*3.1*	*2.2*	*2.5*	*1.6*
**Proteobacteria**	**0.4**	**2.3**	**1.0**	**0.2**
Alcaligenaceae	0.3	2.3	1.0	0.1
*Sutterella*	*0.3*	*2.3*	*1.0*	*0.1*
**Verrucomicrobia**	**5.1**	**14.2**	**11.7**	**6.6**
Verrucomicrobiaceae	5.1	14.2	11.7	6.6
*Akkermansia*	*5.1*	*14.2* *	*11.7* *	*6.6*^ †^

**Phyla**, families, and *genera* with >1% occurrence in the whole population are presented. * *p* < 0.05 vs. cont, ^†^ vs. high-fat (HF), ^‡^ vs. high-fat and high-iron (HFHI), by Bonferroni’s multiple comparison.

## Supplementary Materials

The following are available online at http://www.mdpi.com/2072-6643/10/2/175/s1, Figure S1: Temporal changes of biochemical and liver weight data, Table S1: Necropsy and biochemical data.

## Abbreviations

The following abbreviations are used in this manuscript:

CLD	chronic liver disease
HCC	hepatocellular carcinoma
NAFLD	nonalcoholic fatty liver disease
ROS	reactive oxygen species
NASH	nonalcoholic steatohepatitis
HF	high-fat
HFHI	high-fat and high-iron
HI	high-iron
TBARS	thiobarbituric acid reactive substances
LPS	lipopolysaccharide
HE	hematoxylin and eosin
DAB	3,3′-diaminobenzidine
RT-PCR	real-time reverse transcriptase polymerase chain reaction
ALT	alanine aminotransferase
AST	aspartate aminotransferase
ALP	alkaline phosphatase
T-Bil	total bilirubin
NTBI	non-transferrin-bound iron
