# Supplementary material for: Dietary Iron Supplementation Alters Hepatic Inflammation in a Rat Model of Nonalcoholic Steatohepatitis
Source: Nutrients. 2018 Feb 4;10(2):175. doi: 10.3390/nu10020175 (PMC5852751; doi:10.3390/nu10020175)
Supplement: Supplementary file 1 [file nutrients-10-00175-s001.pdf]

Figure S1. Temporal changes in this model

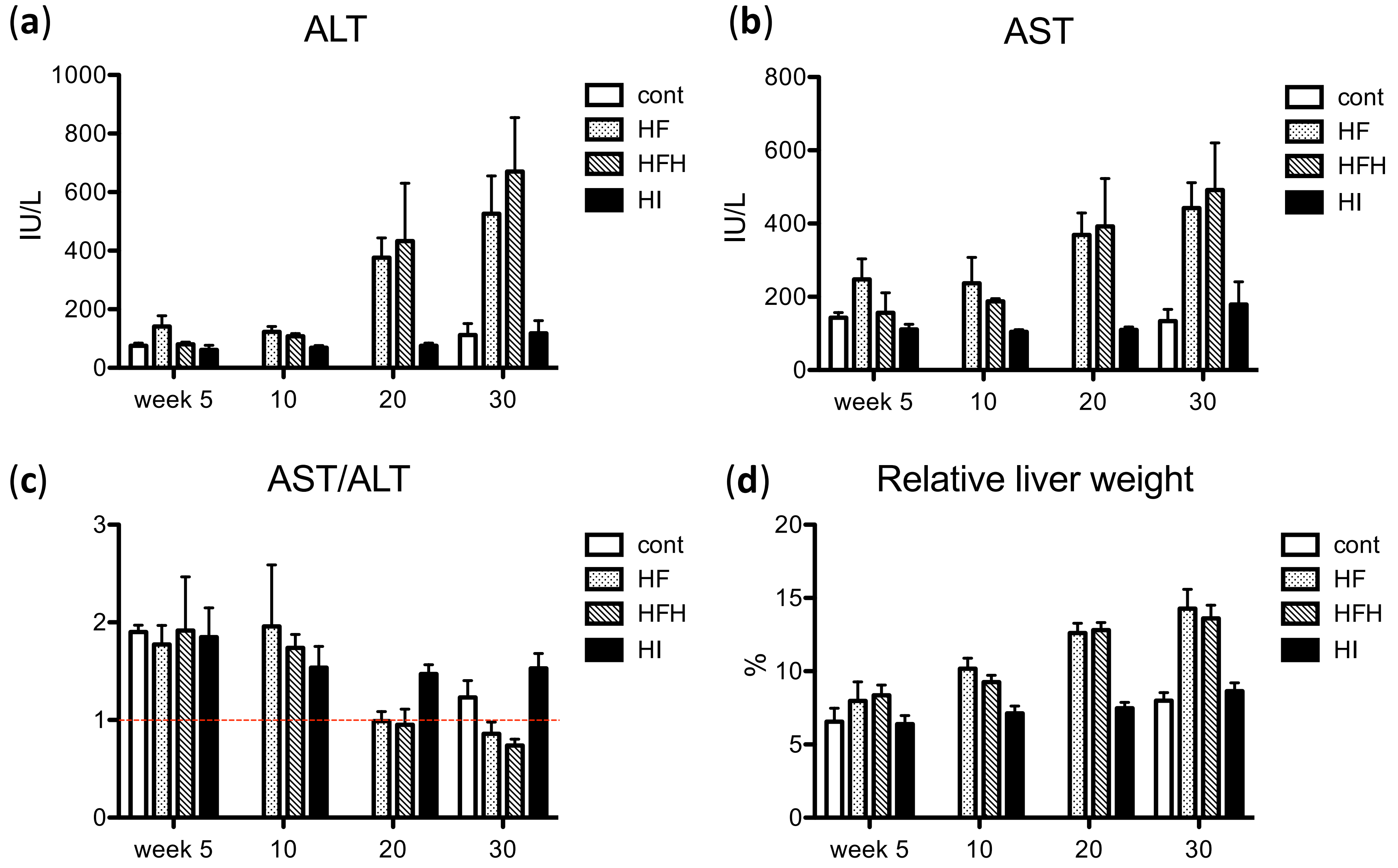

Figure S1. Temporal changes of (**a**) ALT, (**b**) AST, (**c**) ratio of AST/ALT and (**d**) relative liver weight. The red dashed line in (**c**) indicates value 1 of AST/ALT ratio.

Table S1. Necropsy and blood biochemistry data

Time	Group	Animal No.	Body weight (g)	Liver weight (g)	Relative liver weight (%)	ALT (IU/L)	AST (IU/L)	ALP (IU/L)	TBIL (mg/dL)	g-GTP (IU/L)	T-CHO (mg/dL)	TG (mg/dL)	PL (mg/dL)	Glu (mg/dL)	Alb (g/dL)	TP (g/dL)	BUN (mg/dL)	CRE (mg/dL)	Ca (mg/dL)	P (mEq/L)	Na (mEq/L)	K (mEq/L)	Cl (mEq/L)
week 5	Cont	1-1	234	7.4	3.2	74	141	741	0.000	1.82	53	39	85	68	4.2	5.9	27.12	0.30	10.43	9.35	140	5.4	96
		1-2	257	7.3	2.8	73	145	720	0.000	1.34	55	45	86	94	4.3	6.1	26.72	0.33	10.12	7.94	140	4.4	97
		1-3	215	5.7	2.7	67	127	677	0.000	0.76	49	44	85	107	4.4	6.1	23.70	0.32	9.98	7.62	139	4.7	99
		1-4	214	5.8	2.7	88	160	726	0.000	3.28	49	26	81	119	4.3	5.9	24.57	0.28	9.76	7.83	138	4.5	99
	HF	2-1	249	9.8	3.9	NA	NA	NA	NA	NA	NA	NA	NA	NA	NA	NA	NA	NA	NA	NA	NA	NA	NA
		2-2	230	7.6	3.3	112	223	948	0.000	0.70	58	26	59	66	4.2	6.0	22.41	0.30	10.46	6.47	138	5.3	94
		2-3	240	7.8	3.3	182	312	1010	0.000	1.29	76	67	67	68	4.2	5.9	22.09	0.31	10.43	5.41	134	5.2	93
		2-4	217	6.7	3.1	129	208	975	0.002	1.25	67	70	68	113	4.3	5.8	23.08	0.34	9.76	5.79	136	4.6	96
	HFHI	3-1	233	8.1	3.5	NA	NA	NA	NA	NA	NA	NA	NA	NA	NA	NA	NA	NA	NA	NA	NA	NA	NA
		3-2	240	8.7	3.6	83	199	916	0.000	1.74	112	78	105	79	3.8	6.3	18.2	0.35	9.96	6.91	136	5.3	96
		3-3	227	7.5	3.3	86	175	913	0.000	1.67	89	59	83	104	4.1	5.9	17.07	0.32	9.74	6.62	138	4.8	98
		3-4	255	9.1	3.6	72	95	979	0.000	1.05	77	64	91	109	4.6	6.6	19.99	0.38	11.38	9.83	139	5.8	95
	HI	4-1	244	7.2	3.0	84	121	790	0.023	0.96	55	74	104	106	4.7	6.6	20.43	0.33	11.43	10.16	139	5	95
		4-2	228	6.3	2.8	57	123	799	0.001	1.07	54	67	90	79	4.4	6	19.25	0.36	10.41	8.83	141	4.9	97
		4-3	235	6.3	2.7	48	93	606	0.004	1.47	48	52	83	131	4.1	5.6	18.92	0.32	9.68	7.63	139	4.4	100
		4-4	224	5.8	2.6	57	106	749	0.000	2.13	49	65	87	137	4.3	6	19.8	0.28	9.77	8.11	138	4.3	99
week 10	HF	2-1	283	11.0	3.9	119	316	644	0.000	0.00	54	33	61	80	3.9	6.2	18.39	0.34	9.40	6.73	144	5.0	112
		2-2	295	9.8	3.3	144	165	590	0.000	0.00	50	43	62	117	3.9	6.1	18.92	0.4	9.61	5.66	143	4.2	88
		2-3	314	9.7	3.1	101	190	570	0.000	0.00	49	37	57	57	3.9	6.0	16.25	0.31	8.69	6.13	146	4.8	116
		2-4	289	NA	NA	128	276	680	0.000	0.00	82	86	84	88	4.2	6.8	25.18	0.28	8.68	8.12	146	6.0	102
	HFHI	3-1	282	9.5	3.4	113	181	666	0.000	1.63	54	38	64	83	4.1	6.3	18.46	0.38	9.77	8.71	151	4.8	116
		3-2	290	9.2	3.2	114	197	595	0.000	1.11	51	26	56	57	4.0	6.3	19.67	0.31	9.56	6.71	150	5.5	118
		3-3	315	9.7	3.1	95	183	598	0.000	0.00	53	33	63	88	4.2	6.7	15.52	0.34	9.16	5.82	148	4.6	118
		3-4	303	8.6	2.8	111	189	634	0.000	0.00	48	31	57	86	4.2	6.5	15.40	0.34	9.04	6.30	149	4.2	118
	HI	4-1	295	7.1	2.4	62	110	444	0.000	0.72	38	31	66	124	3.8	5.7	23.93	0.31	9.53	6.5	142	4.6	89
		4-2	300	7.7	2.6	66	98	419	0.003	0.76	47	49	84	143	4.1	6.0	25.31	0.33	9.65	6.48	142	4.2	88
		4-3	290	7.2	2.5	77	104	455	0.000	0.00	50	57	87	157	4	6.0	22.03	0.28	9.64	6.69	141	4.4	88
		4-4	297	6.5	2.2	NA	NA	NA	NA	NA	NA	NA	NA	NA	NA	NA	NA	NA	NA	NA	NA	NA	NA
week20	HF	2-1	328	12.3	3.8	299	331	524	0.053	1.06	64	60	82	117	3.9	6.1	20.82	0.33	9.73	3.9	140	4.9	123
		2-2	353	12.1	3.4	352	345	446	0.032	1.69	61	40	71	96	4.1	6.5	19.03	0.33	9.99	4.54	143	4.7	121
		2-3	369	13.6	3.7	458	458	471	0.036	2.66	70	58	86	104	4.1	6.3	19	0.34	9.79	4.51	142	4.9	118
		2-4	349	12.4	3.6	396	343	470	0.000	1.27	64	51	78	114	4	6.1	18.62	0.26	9.75	5.27	140	4.5	116
	HFHI	3-1	366	12.4	3.4	298	320	422	0.000	0.00	59	50	71	107	3.8	6.1	22.37	0.29	9.73	6.2	142	5.6	118
		3-2	386	13.3	3.4	262	289	488	0.034	0.19	88	89	92	127	4.1	6.3	23.3	0.29	9.86	5.42	141	5	115
		3-3	328	12.3	3.7	477	380	485	0.024	2.31	76	45	84	126	3.8	6.1	17.48	0.25	9.28	5.19	139	5	114
		3-4	355	13.2	3.7	694	580	480	0.028	2.09	94	60	92	111	4.1	6.7	19.22	0.3	10.13	5.76	141	5.2	114
	HI	4-1	325	7.6	2.3	81	113	332	0.024	0.00	52	62	88	148	4.1	5.9	22.22	0.27	10.23	4.72	141	4.8	115
		4-2	318	7.0	2.2	61	98	284	0.016	0.00	58	37	90	123	4.2	6.2	21.18	0.19	10.19	5.37	146	4.9	117
		4-3	342	7.8	2.3	76	111	327	0.016	0.00	58	71	96	122	4.3	6.4	28.74	0.42	11.02	6.21	144	5.1	111
		4-4	352	NA	NA	82	117	315	0.000	0.81	61	64	101	66	4.2	6.3	20.52	0.12	10.41	5.79	143	4.6	112
week 30	cont	1-1	345	8.2	2.4	108	148	415	0.007	2.31	68	96	114	170	4.2	6.1	28.09	0.28	9.64	4.89	141	5	108
		1-2	364	8.6	2.4	169	170	310	0.028	2.90	74	60	120	121	4.1	6.2	20.81	0.23	10.08	5.25	141	4.7	106
		1-3	357	7.3	2.0	88	120	315	0.018	0.00	55	49	88	147	4.2	6.1	22.66	0.29	9.68	5.54	143	4.4	107
		1-4	363	7.9	2.2	82	97	281	0.020	0.32	63	33	97	147	3.9	5.8	19.94	0.29	9.55	4.97	141	4.2	105
	HF	2-1	430	15.6	3.6	590	439	638	0.038	2.88	103	51	117	123	4.1	6.6	21.26	0.29	10.17	5.16	141	5.3	103
		2-2	387	13.3	3.4	420	428	655	0.027	6.16	99	44	109	162	3.7	6.1	19.85	0.3	9.7	4.62	141	4.9	104
		2-3	417	15.2	3.6	677	535	534	0.059	1.70	89	39	103	116	3.9	6.2	16.78	0.3	10.49	5.56	143	4.8	105
		2-4	364	13.0	3.6	418	367	506	0.030	1.47	90	37	93	141	3.9	6.1	19.47	0.29	10.03	5.31	141	4.3	102
	HFHI	3-1	360	12.8	3.6	857	637	532	0.069	1.79	112	43	114	128	4.1	6.6	18.1	0.31	10.38	4.7	142	4.1	102
		3-2	415	13.4	3.2	665	447	463	0.070	1.80	87	34	95	118	4	6.5	13.81	0.29	10.16	5.44	144	4.7	104
		3-3	400	14.6	3.7	488	391	516	0.036	2.16	94	33	98	132	3.8	6.1	15.47	0.2	9.4	5.09	141	4.9	101
	HI	4-1	393	9.0	2.3	87	126	344	0.021	0.71	81	74	120	146	4.4	6.7	24.36	0.26	10.38	5.61	141	4.8	101
		4-2	374	8.4	2.2	89	156	295	0.021	0.27	73	79	117	163	4.2	6	24.41	0.29	10.32	5.01	140	4.9	101
		4-3	413	9.2	2.2	180	268	385	0.047	0.91	78	101	129	171	4.3	6.5	21.91	0.25	10.33	4.79	142	4.6	102
		4-4	378	8.0	2.1	117	167	355	0.021	2.97	70	70	111	166	4.1	6.1	20.26	0.27	9.74	4.35	141	4.5	102

NA: not available, ALT: alanine aminotransferase, AST: aspartate aminotransferase, ALP: alkaline phosphatase, TBIL: total bilirubin, g-GTP: gamma-glutamyltransferase, T-CHO: total cholesterol, TG: triglyceride, PL: phospholipid, Glu: glucose, Alb: albumin, TP: total protein, BUN: blood urea nitrogen, CRE: creatinine
